# Aerobic Fitness Level Affects Cardiovascular and Salivary Alpha Amylase Responses to Acute Psychosocial Stress

**DOI:** 10.1186/s40798-016-0057-9

**Published:** 2016-08-23

**Authors:** Thomas Wyss, Maria Boesch, Lilian Roos, Céline Tschopp, Klaus M. Frei, Hubert Annen, Roberto La Marca

**Affiliations:** 1 Swiss Federal Institute of Sport Magglingen (SFISM), Magglingen, Switzerland; 2 Clinical Psychology and Psychotherapy, University of Zurich, Zurich, Switzerland; 3 Military Academy, Swiss Federal Institute of Technology, ETH Zurich, Zurich, Switzerland

**Keywords:** Physical fitness, Physical activity, Stress response, Stress prevention, Autonomic nervous system, Cross-stressor adaptation hypotheses

## Abstract

**Background:**

Good physical fitness seems to help the individual to buffer the potential harmful impact of psychosocial stress on somatic and mental health. The aim of the present study is to investigate the role of physical fitness levels on the autonomic nervous system (ANS; i.e. heart rate and salivary alpha amylase) responses to acute psychosocial stress, while controlling for established factors influencing individual stress reactions.

**Methods:**

The Trier Social Stress Test for Groups (TSST-G) was executed with 302 male recruits during their first week of Swiss Army basic training. Heart rate was measured continuously, and salivary alpha amylase was measured twice, before and after the stress intervention. In the same week, all volunteers participated in a physical fitness test and they responded to questionnaires on lifestyle factors and personal traits. A multiple linear regression analysis was conducted to determine ANS responses to acute psychosocial stress from physical fitness test performances, controlling for personal traits, behavioural factors, and socioeconomic data.

**Results:**

Multiple linear regression revealed three variables predicting 15 % of the variance in heart rate response (area under the individual heart rate response curve during TSST-G) and four variables predicting 12 % of the variance in salivary alpha amylase response (salivary alpha amylase level immediately after the TSST-G) to acute psychosocial stress. A strong performance at the progressive endurance run (high maximal oxygen consumption) was a significant predictor of ANS response in both models: low area under the heart rate response curve during TSST-G as well as low salivary alpha amylase level after TSST-G. Further, high muscle power, non-smoking, high extraversion, and low agreeableness were predictors of a favourable ANS response in either one of the two dependent variables.

**Conclusions:**

Good physical fitness, especially good aerobic endurance capacity, is an important protective factor against health-threatening reactions to acute psychosocial stress.

## Key Points

1. Aerobic fitness level affects cardiovascular responses to acute psychosocial stress.
2. Aerobic fitness level affects alpha amylase responses to acute psychosocial stress.
3. Present results support the cross-stressor adaptation hypothesis, saying that even if psychological coping with stress remains unaffected, the various training-induced adaptations in the organization of the ANS of an aerobically fit person influences physiological responses to acute stress.

## Background

Psychosocial stress has a harmful impact on somatic and mental health [1, 2]. In order to improve individual resistance to psychosocial stress, regular physical activity seems to be an effective strategy [3–7]. This relation is based on similarities between physiological processes during physical and psychological stress [8, 9]. A situation which is perceived as stressful, independent of whether the source is physical [10, 11] or psychological [12, 13], leads to an altered activity of the hypothalamic-pituitary-adrenal (HPA) axis and the autonomic nervous system (ANS). Because regular physical training has been shown to diminish the reactivity of the HPA axis and ANS during exercises [10, 11], a cross-adaptation to psychological stress is assumed [14]. To test the cross-stressor adaptation hypothesis [15] discussed above, many studies investigated the relation between physical activity behaviour and psychological stress reactivity. However, the health-promoting effect of regular physical exercise on stress reactivity is most probably based on a combination of two effects: better psychological resources resulting in improved psychological coping with stress and (2) reduced physiological reactivity to stress [14]. De Geus and Stubbe believe that even if psychological coping with stress remains unaffected, the various training-induced adaptations in the organization of the ANS and its target organs influence the pattern and intensity of physiological responses to stress [14]. To investigate that hypothesis, the influence of physical fitness on stress responses should be investigated while controlling for physical activity behaviour and potential stress-influencing factors. As potential stress-influencing factors, personality- and behaviour-related factors were only selected when prior studies have demonstrated their relation to stress reactions. Some evidence suggests that personality traits, resilience, smoking habits, educational level, and migrating background are influencing perception and responses to psychosocial stress [16–20].

It is the aim of the present study to investigate the role of physical fitness level on ANS (i.e. heart rate and salivary alpha amylase) responses to acute psychosocial stress, while controlling for established factors influencing individual stress reactions. Using the framework of the cross-stressor adaptation in the present study, the hypothesis was that the individual physical fitness level modifies the physiological stress responses to non-exercise stressors. Lower baseline heart rate (HR), stronger HR reaction to non-exercise stressors, faster HR recovery after termination of a non-exercise stressor, and lower salivary alpha amylase (sAA) immediately after the non-exercise stressor are expected in physically well-trained subjects.

## Methods

### Study Design and Participants

Volunteers were recruited among the 693 young men who started their mandatory military service in the Swiss Armed Forces garrison located in Aarau, Switzerland. This sample is representative of the healthy young Swiss male population. In total, 651 recruits (94 %) gave their informed consent to volunteer in the scientific project, approved by the local Ethics Committee of the Canton Aargau, Switzerland (registration number 2011/008). All research was performed in accordance with the ethical standards of the Declaration of Helsinki. For the present study, 302 recruits were randomly selected from the pool of volunteers to take part in a psychosocial stress examination. In the first week of basic military training (BMT), the selected volunteers participated in an acute psychosocial stress test (Trier Social Stress Test for Groups [TSST-G]), while HR was monitored continuously and saliva samples were collected immediately before and after the TSST-G for analyses of sAA. In the same week, all volunteers participated in a physical fitness test and they responded to a battery of questionnaires regarding lifestyle factors and personal traits.

### Stress Provocation

To induce acute psychosocial stress, the TSST-G was applied as stated by Bösch et al. [21] and first described by von Dawans et al. [22]. The TSST-G is an established, standardized performance test protocol that combines a high level of social-evaluative threat and uncontrollability to provoke psychosocial stress [21]. In the present study, four participants were seated next to each other but separated by privacy protection walls. After 2 min of baseline measurement, the upcoming task was introduced and subjects were allowed to prepare themselves for 2 min. Then, a fake expert panel entered the room and turned on video cameras to make the subjects believe that they were being videotaped. Each participant had 2 min to introduce himself for a mock job interview. After completion of the four short interviews, participants were asked to perform a mental arithmetic task consisting of continuous subtraction, as quickly and accurate as possible. After each mistake, subjects had to restart from the beginning. Again, this examination took 2 min for each of the four participants. Finally, the TSST-G was followed by 6 min of recovery measurement.

### Data Collection

Heart rate was continuously measured during the stress examination using an ambulatory electrocardiography system (Equivital System; Hidalgo, Cambridge, UK). Data were edited manually (VivoSense, Vivonoetics, San Diego, CA), and the average HR (in beats per minute [bpm]) was calculated for 2-min time intervals.

Salivary alpha amylase samples were collected immediately before and after the TSST-G. Participants were requested to gently chew on a Salivette (Sarstedt, Sevelen, Switzerland) for 1 min. The samples were stored at −20 °C until analyses were conducted in the biochemical laboratory of the Department of Clinical Psychology and Psychotherapy at the University of Zurich, Switzerland. The activity of sAA was analysed with a kinetic colorimetric test using assay kits from Roche (Roche 11555685 alpha-Amylase Liquid acc, Roche, Basel, Switzerland) and an automatic analyser (Biotek Instruments, Lucern, Switzerland) with the software KC4 (Roche).

Physical fitness was assessed using the Swiss Army physical fitness test battery [23]. The test battery contained a progressive endurance run (PER) to measure aerobic endurance capacity; a standing long jump (SLJ) and a seated shot put (SSP) to measure muscle power of the lower and upper extremities, respectively; a trunk muscle strength test (TMS) to measure trunk muscle fitness; and a one-leg standing test (OLS) to measure balance. The PER is a paced running test, conducted on a 400-m outdoor track according to the protocol developed by Conconi et al. [24] and evaluated using the final running velocity. Based on PER, the maximal oxygen consumption (V̇O_2_max) can be estimated using the formula by Wyss et al. [23]. The SLJ was performed from the gym hall floor onto a mat of 7-cm height. The SSP was performed as a 2-kg ball chest pass while sitting upright on a bench with the back touching a solid wall. In the TMS, the subjects had to hold an isometric body position (prone bridge) [25] for as long as possible, while lifting their feet alternately. In OLS, participants had to keep position on one leg as long as possible while closing their eyes after 10 s and laying their head back while keeping the eyes closed after 20 s. Time was measured for the left and right legs separately, and the sum of both was used as a value for balance ability. Precise descriptions of the five tests were published elsewhere [23]. To assess anthropometry, body height was measured to the nearest 0.1 cm using a stadiometer (Seca model 214, Seca GmbH, Hamburg, Germany) and body weight to the nearest 0.1 kg on a calibrated digital balance (Seca model 877, Seca GmbH, Hamburg, Germany).

Data on personality traits, behavioural factors, and socioeconomic data were assessed by questionnaires. Data on the personality of participants were assessed using the German version of the NEO Psychological Personality Inventory with 240 items and five-point Likert scale by Ostendorf and Angleitner [26]. Participants’ resilience was assessed by the short version of the resilience scale (RS) [27], consisting of 11 items answered on a seven-point Likert scale. The German version of the perceived stress questionnaire (PSQ) [28] with 20 items and a four-point Likert scale was used to detect subjects’ perceived chronic stress level. Physical activity behaviour was assessed using the International Physical Activity Questionnaire (IPAQ short) [29], and participants were classified as inactive, partly active, unregularly active, regularly active, and trained. Further, questions that were previously not validated about smoking habits, migration background, and educational level were used to group participants as smoker and non-smoker and Swiss citizens with and without a migration background and in three categories of educational levels (lower secondary school, upper secondary school, and high school).

### Statistical Analysis

Statistical analyses were performed using SPSS for Windows (version 22.0, IBM, Chicago, IL) with a level of significance of *p* < .05 to indicate statistical significance. Group results are presented as mean ± standard deviation. A multiple linear regression was run to predict ANS responses to acute psychosocial stress from physical fitness test performances, controlling for personal traits, behavioural factors, and socioeconomic data. As a dependent variable representing magnitude of cardiovascular response, the area under the individual HR response curve (AUCg) was calculated using a trapezoid formula [30], as has been done before to represent physiological responses [5]. As a dependent variable representing sympathetic sAA reactivity, the increase from pre- to post-TSST-G as well as the sAA level determined immediately after the TSST-G (i.e. post level) was used. The latter variable was included because baseline levels usually constitute a period of combined sympathetic and, probably more important, parasympathetic activity, and the latter can positively influence amylase activity itself [31, 32]. Therefore, it is unclear which of both variables, the change value or the value determined after the stress test, better reflects sympathetic stress reactivity. The potential independent predicting variables are listed in Table 1. Regression analyses were performed twofold, with the same result: by backwards elimination and by systematic entering of the variables in the order of the strength of the association with the dependent variable. Variables which did not predict the dependent variable significantly (*p* > .05) were removed from the model. The assumptions of linearity, non-collinearity, independence of errors, homoscedasticity, unusual points, and normality of residuals were met. The effect size of predicting variables was calculated according to Cohen [33] as ${f}^2=\frac{\left({R}_{\;\mathrm{including}}^2-{R}_{\;\mathrm{excluding}}^2\right)}{1-{R}_{\;\mathrm{including}}^2}$. Cohen termed an effect size of .02, .15, and .35 as small, medium, and large, respectively. Fitness variables remaining in the final regression model were used to stratify volunteers in four performance groups of a similar number of participants for each performance test. For example, the 25 % of participants with the best PER performances were stratified to the first quartile, representing volunteers with highest aerobic fitness level, while the fourth quartile represented those 25 % with the lowest aerobic fitness level. HR reactivity was calculated by the absolute difference in HR values in the 2-min segment before the TSST-G and the 2-min segment of the interview task during the TSST-G (first HR-peak). HR recovery was calculated by the absolute difference in HR values in the 2-min segment of the mental arithmetic task (second HR-peak) and the 2-min segment starting 6 min after the end of the TSST-G. Group comparisons were conducted using independent sample *t* tests. Furthermore, repeated measures ANOVA was used to investigate effects of group, time, and group × time interaction on HR and sAA levels during TSST-G. In all the presented data, the Mauchly test showed that the assumption of sphericity has been violated; therefore, the Greenhouse-Geisser estimate was used to report repeated measures ANOVA results.

**Table 1:** Potential predicting variables for ANS stress reactivity

Physical fitness	Volunteers’ mean values
Aerobic fitness by progressive endurance run (V̇O_2_max)	50.1 ± 5.0 ml/kg/min
Muscle power of the lower extremities by standing long jump	2.3 ± 0.2 m
Muscle power of the upper extremities by seated shot put	6.4 ± 0.7 m
Trunk muscle fitness by trunk muscle strength test	133.3 ± 64.0 s
Balance by one-leg standing test	47.1 ± 9.9 s
Anthropometry by body mass index	23.6 ± 3.0 kg/m^2^
Covariates	Volunteers’ mean values or proportion
Physical activity behaviour by IPAQ short (index)	59.4 % regularly active or trained
Personality by NEO	
Neuroticism (index 0–192)	76 ± 19
Extraversion (index 0–192)	118 ± 20
Openness for experiences (index 0–192)	108 ± 16
Agreeableness (index 0–192)	108 ± 16
Conscientiousness (index 0–192)	117 ± 20
Perceived stress by PSQ (index 0–1)	0.3 ± 0.2
Resilience by RS short (index 0–77)	60 ± 10
Smoking status (yes/no)	34.0 % yes
Migration background (yes/no)	18.9 % yes
Educational level (low, medium, high)	31.3 % low, 41.8 % medium, 26.9 % high

*ANS* autonomic nervous system

## Results

### Participants

Complete data on 219 male recruits (73 % of the total sample of selected volunteers) with a mean age of 20.2 ± 1.1 years, a body size of 177.8 ± 6.6 cm, and a body mass of 74.9 ± 11.3 kg were registered. Volunteers’ mean values and proportions for all independent variables are presented in Table 1. Participants’ V̇O_2_max was significantly correlated to the SLJ, and SSP performance (*r* = .190 and −.190, *p* < .01, respectively), while the two latter ones were positively correlated to each other as well (*r* = .180, *p* = .007). Further, participants’ V̇O_2_max was significantly correlated to their physical activity and smoking behaviour as well as to their educational level (*r* = .163, −.186, and .170, *p* < .05, respectively), and their smoking habit was correlated with their educational level and agreeableness (*r* = .347, and .236, *p* < .001, respectively). Participants that were classified as insufficiently physically active (less than 150 min of moderate or 75 min of vigorous physical activities per week) had a lower V̇O_2_max level, compared to the participants who were sufficiently physically active (48.87 ± 4.46 and 50.99 ± 5.10 ml/kg/min, *p* = .002).

### Cardiac Response to Acute Stress

A multiple linear regression predicting HR-AUCg revealed performances at the PER, at the SLJ, and smoking attitude as significant independent variables (Table 2). The many other covariates—BMI, physical activity behaviour, resilience, personality traits, educational level, and migration background—proved statistically insignificant. The assumptions of linearity, non-collinearity, independence of errors, homoscedasticity, unusual points, and normality of residuals were met by the used model (Table 2). The variables in the final regression model significantly predicted HR-AUCg, *F* = 12.626, *p* < .001, adj. *R*^2^ = .150. Regression coefficients, kept on measurement scale, standard errors, and their respective 95 % confidence intervals (CIs) can be derived from Table 2.

**Table 2:** Summary of multiple regression analysis of HR-AUCg

Variable	*B*	SE	*β*	*t*	Sig	CI (95)	
Intercept	1573.776	257.158		6.120	0.000	1066.903	2080.649
Progressive endurance run (s)	−0.447	0.087	−0.336	−5.145	0.000	−0.618	−0.276
Standing long jump (m)	420.851	112.601	0.240	3.738	0.000	198.908	642.793
Smoking^a^ (0 = no; 1 = yes)	−114.471	40.811	−0.180	−2.805	0.005	−194.913	−34.030

*HR-AUCg* area under the HR response curve, *B* unstandardized regression coefficient, *SE* standard error of the coefficient, *β* standardized coefficient, *Sig* significance, *CI (95)* 95 % confidence interval

^a^Smoking status before military service

**Fig. 1:**
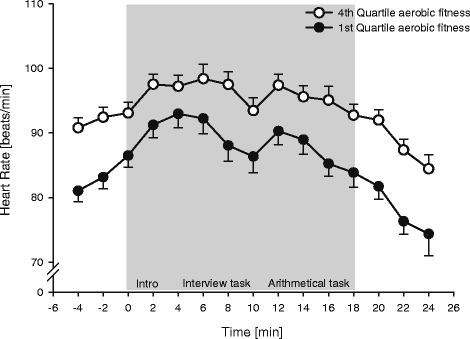
HR before, during, and after the TSST-G among volunteers of high and low aerobic fitness. Values are presented as mean ± SEM for each 2-min time interval. The first quartile represents subjects of high aerobic fitness; the fourth quartile represents subjects of low aerobic fitness. Each of the 15 time-segments in the figure represents data from all 219 subjects. *HR* heart rate, *TSST-G* Trier Social Stress Test for Groups

The strongest influence on total magnitude of HR response to acute stress was found for aerobic fitness (Cohen’s effect size of *f*^*2*^ = .12). Participants were, therefore, stratified into four groups of aerobic fitness, with 55 volunteers in the first quartile of aerobic fitness (V̇O_2_max of 55.8 ± 1.6 ml/kg/min) and 54 volunteers in the fourth quartile (V̇O_2_max of 43.3 ± 2.6 ml/kg/min). The HR responses to the TSST-G in these groups are presented in Fig. 1. As Fig. 1 indicates, a repeated measures ANOVA showed a significant main effect on HR by the factors group (*F* = 10.622, *p* = .002) and time (*F* = 19.271, *p* < .001), but not by the interaction between group and time (*F* = 1.618, *p* = .137). HR values were significantly different (*p* < .05) between groups at baseline. However, HR reactivity in participants of high aerobic fitness level was stronger (+11.9 ± 10.1 bpm) than that in participants of low aerobic fitness level (+6.4 ± 9.1 bpm, *p* = .004). HR recovery was not different between the two groups (−11.3 ± 10.8 and −9.4 ± 7.2 for first and fourth quartiles, respectively, *p* = .370).

**Fig. 2:**
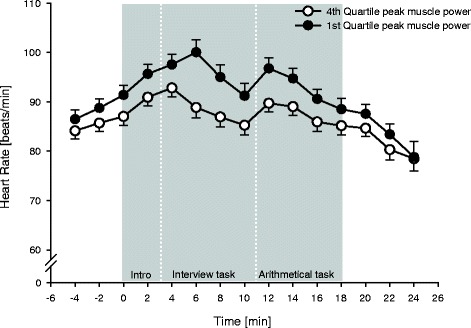
HR before, during, and after the TSST-G among volunteers of high and low muscle power in the lower extremities. Values are presented as mean ± SEM for each 2-min time interval. The first quartile represents subjects of high muscle power. The fourth quartile represents subjects of low muscle power. Each of the 15 time-segments in the figure represents data from all 219 subjects. *HR* heart rate, *TSST-G* Trier Social Stress Test for Groups

The same comparison, using SLJ performance to stratify volunteers in different groups, resulted in Fig. 2. The 52 volunteers in the first quartile of the lower extremity muscle power had a better SLJ performance (2.5 ± 0.1 m) than that of the 56 volunteers in the fourth quartile (2.1 ± .1 m). The HR response to the TSST-G in those two SLJ performance groups is presented in Fig. 2. As Fig. 2 indicates, a repeated measures ANOVA showed a significant main effect on HR by the factors group (*F* = 4.469, *p* = .037) and time (*F* = 24.560, *p* < .001) as well as the interaction between group and time (*F* = 3.259, *p* = .003). HR values of the two performance groups were not different before and after the TSST-G intervention. However, HR reactivity in the first quartile of SLJ performances was stronger (+13.5 ± 13.3 bpm) than that in participants of the fourth quartile of SLJ performances (+4.8 ± 9.8 bpm, *p* = .001). HR recovery was not different between the two groups (−12.0 ± 9.4 and−9.8 ± 8.2 for the first and fourth quartiles, respectively, *p* = .221).

Smokers and non-smokers had the same baseline and recovery HR before and after the TSST-G. However, non-smokers showed a stronger HR reaction to the TSST-G (+10.5 ± 9.7 bpm) compared to smokers (+6.0 ± 7.6, *p* = .001).

Subjects’ HR-peak levels during the interview task (first HR-peak) and the mental arithmetic task (second HR-peak) were comparable (93.2 ± 16.4 and 92.2 ± 14.6 bpm, *p* = .166). However, HR during those 2-min time intervals were higher (*p* < .001) than the task-specific average HR over 8 min (91.6 ± 14.4 bpm during interview task and 89.7 ± 13.2 bpm during arithmetic task).

### sAA Response to Acute Stress

A multiple linear regression predicting the change in salivary alpha amylase (ΔsAA) values (increase in sAA level from pre- to post-TSST-G) revealed performance at the OLS (*B* = .764, SE = .313, *β* = .170, *t* = 2.437, *p* = .016) and educational level (*B* = 13.079, SE = 4.127, *β* = .223, *t* = 3.169, *p* = .002) as the only significant independent variables. The variables in the final regression model explained 5 % of the variance in the ΔsAA values, *F* = 2.825, *p* = .012, and adj. *R*^2^ = .050.

A multiple linear regression predicting the sAA stress response to acute stress (sAA level after TSST-G) revealed performance at the PER, performance at the OLS, and the two personality traits “extraversion” as well as “agreeableness” as relevant independent variables (Table 3). The variables in the final regression model significantly predicted the sAA stress response, *F* = 8.796, *p* < .001, and adj. *R*^2^ = .124. Regression coefficients, kept on measurement scale, standard errors, and their respective 95 % CIs can be derived from Table 3.

**Table 3:** Summary of multiple regression analysis of the sAA stress response

Variable	*B*	SE	*β*	*t*	Sig	CI (95)	
Intercept	−23.049	35.859		0.644	0.520	−47.579	93.778
Progressive endurance run (s)	−0.048	0.017	−0.182	−2.821	0.005	−0.081	−0.014
One-leg standing test (s)	1.538	0.393	0.249	3.909	0.000	0.762	2.313
Extraversion (index)	−0.496	0.197	−0.163	−2.524	0.012	−0.884	−0.109
Agreeableness (index)	0.702	0.228	0.200	3.080	0.002	0.253	1.150

*sAA* salivary alpha amylase, *B* unstandardized regression coefficient, *SE* standard error of the coefficient, *β* standardized coefficient, *Sig* significance, *CI (95)* 95 % confidence interval

**Fig. 3:**
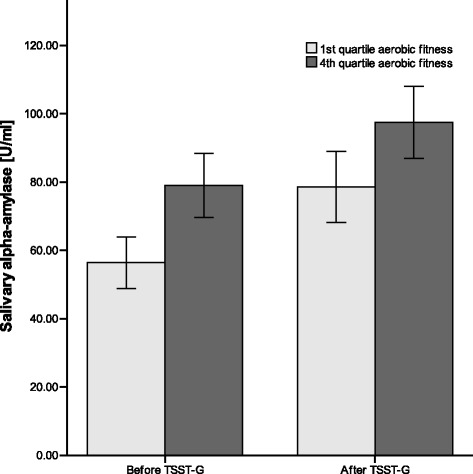
Salivary alpha amylase stress response in subjects of high and low aerobic fitness. Values are presented as mean ± SEM. Salivary alpha amylase was investigated immediately before and after the Trier Social Stress Test for Groups (TSST-G). The first quartile represents subjects of high aerobic fitness. The fourth quartile represents subjects of low aerobic fitness

Aerobic fitness was the only variable represented in the models, predicting HR and sAA responses to acute stress. Cohen’s effect size for PER as a predictor variable for the sAA stress response was *f*^2^ = .03. The sAA values immediately before and after the TSST-G of recruits in the quartile of the lowest and highest aerobic fitness level are presented in Fig. 3. A repeated measures ANOVA with time (before and after TSST-G) as within-subjects factor and group affiliation as between-subjects factor showed a significant main effect on sAA values by the factors group (*F* = 10.369, *p* = .002) and time (*F* = 16.434, *p* < .001), but not for the interaction between group and time (*F* = 0.474, *p* = .492).

## Discussion

HR and sAA responses to acute psychosocial stress were shown to be dependent on recruits’ physical fitness level, even when controlled for physical activity behaviour and further relevant covariates. Therefore, the present study supports the hypothesis of De Geus and Stubbe [14] that the various fitness-related adaptations in the organization of the ANS and its target organs influence the pattern and intensity of physiological responses to stress, even if psychological coping with stress remains unaffected. HR-AUCg was predicted by performances at the PER, SLJ, and regular smoking behaviour (Table 2). sAA stress responses were predicted by performance at the PER and OLS, as well as by values representing the personality trait “extraversion” and “agreeableness” (Table 3). Surprisingly, physical activity behaviour did not remain in the regression models, and only a weak relation to aerobic fitness was demonstrated (*r* = .163). That might be due to the methodological disadvantages of self-reported questionnaires to assess physical activity [34]. In the following sections, the relationship of each predicting variable to HR-AUCg and sAA is discussed and compared to results of previous studies.

### Cardiac Response to Acute Stress

The cumulative HR-AUCg value, as an index of the total HR output across the measurement period, has been shown to be helpful to detect relevant variables, predicting cardiac response to acute stress [5]. However, HR-AUCg is strongly dependent on three aspects of HR development, on baseline HR, HR reactivity during TSST-G, and on HR recovery after TSST-G. A healthy HR reaction to stress can be described as low baseline HR, strong HR reaction to acute stress, and fast HR recovery after the acute stress situation [35]. A low baseline or resting heart rate was found in the review of Cooney et al. [36] to be strongly related to reduced incidence of cardiovascular diseases. A meta-analysis about the relation between cardiovascular responses to acute stress and health revealed that a greater reactivity to stress was associated with poor cardiovascular health status [37]. However, included studies usually considered changes in systolic and diastolic blood pressure as a marker for cardiovascular responses. Studies examining HR reaction to acute stress, instead, showed higher increases in HR during acute stress in healthy subjects. For example, in the study of Jackson et al. [13], subjects with lower risk of hypertension showed a HR increase of 20 bpm during mental arithmetic task compared to their peers with higher risk of hypertension with a HR increase of 12 bpm. This observation can be explained by the evolutionary importance to get ready to fight or flight as soon as a threat appears. Furthermore, with regard to the law of initial values (assuming that the magnitude of responses is dependent of the initial baseline level, i.e. a high baseline is associated with a reduced reactivity), one could expect a higher HR reactivity in subjects with lower baseline levels [38]. Nevertheless, further evidence arose showing a decreased HR reaction to acute stress to be related with worse health [35, 39, 40]. Therefore, one has to assume that both exaggerated as well as blunted cardiovascular reactivity can be maladaptive [41, 42]. Finally, a fast HR recovery from acute stress was consistently associated with reduced cardiovascular risk status [37].

A good aerobic endurance capacity is related to healthy cardiac reaction to acute stress. Two out of three criteria of a healthy HR reaction on stress were demonstrated in volunteers in the first quartile of PER performance. They showed a baseline HR of 10 bpm lower than that of volunteers in the fourth quartile of PER. Further, they revealed a significantly greater HR reactivity to acute stress compared to the lowest quartile of cardiovascular fitness. A meta-regression analysis of 73 studies investigating laboratory stress came to the same conclusion that cardiorespiratory fitness is related to greater reactivity and better recovery of HR responses [43]. In contrast, other meta-analyses found a decreased psychological stress reactivity in relation with physical fitness (e.g. Forcier et al. [44]), contributing to the inconsistency in the literature.

Based on the present data, muscle power might be related to a healthy cardiac reaction to acute stress as well (strong HR reactivity and fast HR recovery). Subjects of low and high SLJ performance showed the same baseline HR before the stress task. However, during the TSST-G, the group of subjects with high SLJ performance showed a stronger HR reaction, and a trend of faster HR recovery (Fig. 2), resulting in larger HR-AUCg values. It can be concluded that subjects with high muscle power showed two out of three criteria of a healthy reaction to stress (strong HR reactivity and fast HR recovery) [35] compared to subjects with low muscle power. Based on prior studies on stress perception, we conclude that higher muscle power might lead to healthier physiological reactions to stress as well as to lower perceived stress level as well [45–47]. However, to the knowledge of the authors, the relation between anaerobic fitness, or muscle strength, and physiological responses to non-training-related, acute psychosocial stress is not sufficiently investigated yet. Therefore, further studies in this field are necessary to discuss the results demonstrated in the present study.

Smoking is related to an unhealthy cardiac reaction to acute stress. In agreement with our results, Child and Wit [48] demonstrated smaller relative HR reactivity during a TSST for smokers, compared to non-smokers. Therefore, the ability to adequately react to a stressor seems reduced in smokers. Authors of prior studies suggest that smoking causes a transient but also a long-term reduction in vagal cardiac control in young people [48, 49].

The many other covariates—BMI, physical activity behaviour, resilience, personality traits, educational level, and migration background—proved statistically insignificant in the multivariate regression analysis of the present study.

### sAA Response to Acute Stress

Nater and Rohlender [50] demonstrated in their review that the saliva enzyme sAA is a sensitive biomarker for stress-related changes in the body that reflects the activity of the sympathetic nervous system. Therefore, they emphasized that sAA level is a good marker to represent stress reactions. Balodis et al. [51] demonstrated a significant positive relation between subjective stress perception (anxiety reactivity) and the increase in sAA from pre- to post-TSST-G as well as the level of sAA after the TSST-G. In conclusion, high ΔsAA and high post-sAA values are related to increased perception of threat in a stressful situation.

A good aerobic endurance capacity might be positively related to healthy sAA reactivity on acute stress. A repeated measures ANOVA analysis demonstrated aerobic fitness-group affiliation to be a significant main effect on sAA, with low physical fitness related to increased sAA values. This result was previously observed in other studies and explained by the cross-stressor adaptation hypothesis. According to the cross-stressor adaptation hypothesis, in physically trained individuals, lower sAA responses to stressors other than the training exercise can be expected [15, 52].

In the present study, volunteers of high-balance ability (assessed by OLS) showed higher ΔsAA values over the acute stress period as well as higher sAA values after the TSST-G. No similar results of other studies exist. However, this positive relation was not expected and does not go in line with the results of Nedeljkovic et al. [53].

Educational level might be related to stress reactivity as well. However, the results of different studies are not consistent. In the present study, a positive relation between educational level and ΔsAA response on acute stress was found. In accordance with our study, Fiocco et al. [54] found higher cortisol reactivity on acute stress in subjects with high educational level. In contrast to our results, a study on stress perception derived reduced perceived stress level in subjects of higher education [20].

Extraversion and agreeableness remained in the regression model, predicting sAA level after TSST-G. The present results indicate that high extraversion leads to reduced sAA reactivity, while high agreeableness leads to increased sAA values after the TSST-G. Therefore, high extraversion and low agreeableness are favourable personality traits in terms of favourable reaction to acute psychosocial stress. Prior studies showed the same relation between extraversion and perceived psychological stress [16, 55]. However, these studies showed no significant relation between agreeableness and perceived psychological stress.

### Relation Between Physical Activity, Physical Fitness, and Stress Reactivity

Regular participation in physical activity (e.g. exercise) is expected to influence strength, balance, and aerobic capacity, three aspects of physical fitness [34]. Exercise results in acute and chronic changes in central and autonomic nervous system activity, the contracting muscles, and the heart, with several mechanisms being involved, including the exercise pressor reflex, arterial and cardiopulmonary baroreceptors, and the central command (for an overview see Fisher et al. [56]). As addressed by the cross-stressor hypothesis [15], physical activity, exercise training, and physical fitness are related to increased parasympathetic and decreased sympathetic activity, myocardial hypertrophy, increased stroke volume, reduced blood pressure during rest, and an increase in muscle capillary density and force [57]. Additionally, beneficial effects were reported with regard to mental health (e.g. depression and anxiety disorders) [58], cognitive function, and the central nervous system, with greater white and grey matter volume [59–61], mainly in the hippocampus and prefrontal cortex (PFC) [34]. Furthermore, an inverse association with central [62] and cardiac oxidative stress [63] and inflammation [64] was found. All together, these findings support the health-protecting and health-promoting effect of physical activity.

When comparing brain and peripheral structures and mechanisms affected by exercise, a wide overlap with structures involved in stress reactivity becomes evident. In fact, the neurophysiological model introduced by Lovallo [42] assumes that differences in stress reactivity derive from differences in three organizational levels. Level I includes the prefrontal cortex (PFC) and the limbic system. Level II addresses the hypothalamus and the brain stem consisting of output signals to the body and feedback to level I. Level III includes the peripheral tissues, with difference reflecting among other things’ variations in autonomic or endocrine output.

Therefore, exercise (or fitness), which is related to better mood and PFC characteristics, seems to show higher activity in level I, eliciting stronger inhibitory control over limbic structures crucial in the control of cardiac activity [65]. When confronted with stressors, the top-down modulation from the PFC to the central nucleus of the amygdala is disinhibited, resulting in multiple mechanisms being activated or inhibited (level II) leading to an increase in HR (level III). These mechanisms include direct activation as well as disinhibition of sympathoexcitatory neurons in the rostral ventrolateral medulla, and inhibition of the nucleus of the solitary tract with subsequent inhibition of vagal motor outputs in the nucleus ambiguous and dorsal vagal motor nucleus [65]. This results in an increase of sympathetic and a decrease in parasympathetic activity and, therefore, a rise in HR. In subjects with higher physical fitness, one can assume higher inhibitory control from the PFC and, therefore, higher parasympathetic and lower sympathetic activity during rest. Nevertheless, when stressed, a fitter subject has the potential to show a larger disinhibition compared to a less fit subject, since the latter probably experiences less inhibition by the PFC already under rest, limiting the capacity to further inhibit the activity of the limbic system. As a consequence of the larger disinhibition in fitter subjects, the net reduction in vagal and increase in sympathetic activity can be larger, allowing for a stronger HR reactivity to stress. These considerations might explain our findings of lower HR under rest and stronger HR stress reactivity in participants with strong aerobic fitness.

### Limitations

In order to measure the genuine stress reactivity of recruits before the influence of any training, reactivity to the TSST-G had to be assessed during their first week of BMT. Since we had only 5 days to investigate volunteers’ stress reactivity, it was not possible to perform the time-consuming TSST-G with all the 651 volunteers. However, we were able to investigate 302 randomly chosen volunteers.

Due to organizational reasons, we were not able to provide 60 min to adapt participants to the laboratory setting, as recommended for the assessment of psychoneuroendocrine baseline data [51]. Unfortunately, due to the short adaptation period, some subjects probably did not reach a calm resting state of mind when sAA baseline data was assessed immediately before the TSST-G.

Salivary samples were only taken twice. More sAA samples before, during, and after the TSST-G would have been more meaningful. Possibly, the peak in sAA response was not assessed due to our study design. However, more salivary samples would have distracted participants from the situation, causing a psychosocial stressful situation.

The order of active participation of the four subjects during the mock job interview and mental arithmetical task was not recorded. In future studies, this information should be recorded in order to compare physiological reactions while actually performing the stress-inducing task and while passively attending and observing the others being stressed.

The present study group does only represent young, healthy men. Our results cannot be generalized per se for women, older men, and physically or mentally ill populations.

## Conclusions

The present study demonstrates that good physical fitness—especially, good aerobic endurance capacity—is an important protective factor against health-threatening reactions to acute psychosocial stress. Cardiovascular fitness remains a significant predictor variable, even when controlled for the most important influencing factors to individual stress reactions. These results have important implications for individuals but also organizations providing mentally and physically demanding jobs. Employers should support and offer access to physical endurance training for their staff, especially in settings where employees regularly have to cope with stressful situations. In conclusion, regular and good physical training of individuals and employees to increase their cardiovascular fitness is not only important for their physical health but also their ability to adequately react to acute psychosocial stress situations.

## Abbreviations

ANS: Autonomic nervous system
AUCg: Area under the curve
BMT: Basic military training
HPA: Hypothalamic-pituitary-adrenal axis
HR: Heart rate
IPAQ: International Physical Activity Questionnaire
OLS: One-leg standing test
PER: Progressive endurance run
PFC: Prefrontal cortex
PSQ: Perceived stress questionnaire
RS: Resilience scale
sAA: Salivary alpha amylase
SLJ: Standing long jump
SSP: Seated shot put
TMS: Trunk muscle strength test
TSST-G: Trier Social Stress Test for Groups
V̇O_2_max: Maximal oxygen consumption
ΔsAA: Change in salivary alpha amylase
